# Respiratory and Related Comorbidities’ Role in the Risk of Acute Sinusitis: A 15-Year Longitudinal Clinical Study

**DOI:** 10.3390/jcm15020660

**Published:** 2026-01-14

**Authors:** Omar Abdel-Fattah Ahmed, Amr Sayed Ghanem, Marianna Móré, Attila Csaba Nagy

**Affiliations:** 1 Department of Epidemiology, Faculty of Health Sciences, University of Debrecen, 4028 Debrecen, Hungary; dr.omar@mailbox.unideb.hu (O.A.-F.A.); aghanem@etk.unideb.hu (A.S.G.); 2 Department of Gerontology, Faculty of Health Sciences, University of Debrecen, 4028 Debrecen, Hungary; more.mariann@etk.unideb.hu

**Keywords:** respiratory, comorbidities, sinusitis, infection, allergic rhinitis, asthma, COPD, cohort, clinical, epidemiology

## Abstract

**Background/Objectives**: Acute sinusitis (AS) is a common infection of the upper respiratory tract that places considerable clinical and economic burden worldwide. Although frequently encountered in practice, the factors that predispose individuals to AS remain poorly understood. This study examined how different respiratory disorders and comorbidities influence the likelihood of developing AS, aiming to clarify its underlying risk profile. **Methods**: A longitudinal analysis was performed using electronic health records from the Clinical Center of the University of Debrecen Hospital. The study cohort (2007–2022) encompassed 37,164 observations. To evaluate the risk of AS progression, Log-Rank tests and Cox proportional hazards regressions were applied whilst adjusting for covariates. **Results**: The risk of developing AS was significantly higher among individuals with preceding respiratory conditions. Patients with common cold demonstrated a 2.3-fold increased risk of developing AS (95% CI [1.51–3.40]). Compared to those without such disorders, participants with acute bronchitis had a 2.5-fold higher hazard of AS (95% CI 1.90–3.26). The strongest association was observed for allergic rhinitis (HR = 4.04, 95% CI 3.18–5.13), followed by chronic sinusitis (HR = 3.10, 95% CI 2.13–4.51). Chronic obstructive pulmonary disease was also identified as a significant predictor for AS (HR = 1.62, 95% CI 1.04–2.52), whereas dental pathologies were associated with a modest protective effect (HR = 0.69, 95% CI 0.48–0.97). **Conclusions**: Patients with allergic rhinitis, chronic sinusitis, acute bronchitis, common cold, or chronic obstructive pulmonary disease have a markedly higher risk of developing AS. Clinicians should actively screen for these conditions when assessing patients with recurrent or severe sinus infections. Early recognition and management of underlying respiratory disorders may reduce AS episodes, promote symptom control, and lessen healthcare burdens. Future research should concentrate on precision medicine to leverage AS preventive and management strategies.

## 1. Introduction

Acute sinusitis (AS) is a common upper respiratory tract disorder that burdens the world’s healthcare systems. Annually, over 20 million affected cases of AS are diagnosed globally across all age groups [1]. This prevalent condition affects approximately 16% of the adult population and accounts for nearly 12 million healthcare provider visits each year [2]. Furthermore, it contributes to an estimated $11 billion in annual healthcare expenditures [3]. AS is among the leading causes of antibiotic prescriptions worldwide, including in the United States [4]. It ranks as the fifth most dominant condition for which antibiotics are prescribed, with over 20% of adult antibiotic prescriptions attributed to sinusitis [5]. In light of recent guidelines, increasing public health concerns regarding antimicrobial resistance, and the necessity for judicious antibiotic use, it is imperative to develop clear and reliable mitigation algorithms for this widespread diagnosis [6]. Given the clinical nature of AS diagnosis, 1–5% of the adult population in Europe is affected by this recurrent disease every year [7].

AS is characterized by inflammation of the paranasal sinuses, typically resulting from viral, bacterial, or, less commonly, fungal infections, and is frequently accompanied by symptoms such as nasal obstruction, facial pain or pressure, and purulent nasal discharge; in severe cases, additional malaise and fever lasting up to four weeks may occur [8].

In Europe, AS is a leading source of primary care consultation, contributing substantially to healthcare expenditures and productivity losses [9].

Despite being highly prevalent, the determinants of AS remain insufficiently characterized, particularly regarding the influence of pre-existing respiratory conditions and systemic comorbidities within regional populations such as Hungary.

Similar to numerous other European nations, Hungary experiences a significant burden of respiratory diseases due to factors such as air pollution, smoking, and exposure to seasonal allergens [10]. According to the recent publications from the European Centre for Disease Prevention and Control, Hungary (35.9%) surpasses the majority of EU/EEA countries (median: 21.8%) in terms of resistance to antimicrobial agents [11].

Respiratory diseases, including common cold, allergic rhinitis, asthma, and chronic sinusitis, are recognized for predisposing individuals to recurrent respiratory infections. Of particular note is the prevalence of allergic rhinitis, often represents a clinical challenge due to its different response to medical treatment, stands as one of the most common respiratory conditions globally. Its severity is classified as either mild or moderate to severe, contingent upon the extent to which it disrupts normal sleep, daily activities, and work performance. National epidemiological studies have documented an increasing prevalence of allergic rhinitis in Hungary [12,13]. Allergic rhinitis is associated with nasal mucosal inflammation and impaired mucociliary clearance, creating circumstances favorable for sinusitis [14,15]. Similarly, chronic sinusitis, characterized by persistent inflammation, is often tied to acute exacerbations due to the accumulation of pathogenic microorganisms [16]. Comorbidities such as asthma, chronic obstructive pulmonary disease, and dental pathologies, including diseases of the hard tissues of the teeth, may exacerbate systemic inflammation or act as reservoirs for other infections, thereby elevating the risk of AS [9].

In both clinical and public health contexts, it is vital to identify individuals at an early stage of AS risk and implement strategies to delay the onset of respiratory complications. Developing personalized interventions is fundamental to reduce the incidence and severity of AS episodes across diverse populations.

Despite the substantial burden of AS, few longitudinal cohort studies have systematically evaluated how a wide spectrum of upper and lower respiratory conditions and related comorbidities jointly influence the time to first AS episode in clinical practice, particularly in Hungary and across Europe.

Previous studies have mainly examined isolated risk factors or were conducted in relatively small cohorts, which may limit the broader applicability of their findings [17,18]. This gap in literature highlights the need for more comprehensive analyses using larger clinical datasets to better identify consistent risk factors and improve prediction of AS [19].

To that end, the present study utilized health records from the Clinical Center of the University of Debrecen, Hungary [20], to perform a retrospective time-to-event analysis. Over a multi-year follow-up, hazard ratios were estimated for AS in relation to selected respiratory conditions and systemic comorbidities, with the aim of identifying clinically relevant risk associations. The overarching objective of our research was to identify those conditions strongly correlated to AS in routine clinical settings, thereby informing risk stratification, providing actionable insights, and guiding the development of more targeted strategies for prevention and management in different contexts.

## 2. Materials and Methods

### 2.1. Study Design and Data Collection

This investigation employed a retrospective longitudinal methodology as a secondary analysis, drawing on a de-identified and anonymized real-life database, to examine the influence of upper and lower respiratory factors together with associated comorbidities on the risk of developing AS in clinical practice. The study encompassed a 15-year follow-up period from 2007 to 2022. Participants were patients from the Clinical Center of the University of Debrecen Hospital, a large tertiary healthcare facility in Hungary, reflecting nuanced hospital-based specialist diagnoses (including otorhinolaryngology) rather than primary care.

Data were extracted from the hospital’s electronic health records database utilizing standardized diagnostic codes from the International Classification of Diseases, Tenth Revision (ICD-10) [21].

The dataset comprised 37,164 records containing ICD-10 diagnostic codes alongside age and gender information.

The cohort included patients who had at least two recorded healthcare encounters and available ICD-10 data on respiratory or related comorbid conditions between 1 January 2007 and 31 December 2022. A minimum of one year of follow-up after the first eligible diagnosis was required to ensure sufficient observation time. Individuals with incomplete data or a prior acute sinusitis (ICD-10 code: J01) diagnosis before baseline were excluded. The primary outcome was the first recorded episode of AS during follow-up. Observation time was measured from the date of the first qualifying diagnosis until AS onset, last recorded encounter, or study end.

### 2.2. Variables of Interest

The study analyzed variables including demographic factors, respiratory diseases, and other comorbidities. Gender was classified as a binary variable (male or female), while age was recorded as a continuous variable in years. Respiratory and related comorbid conditions were defined as binary variables (absent vs. present) using the relevant ICD-10 codes.

The predictor conditions incorporated acute nasopharyngitis [common cold] (ANP), acute pharyngitis (AP), acute tonsillitis (AT), acute laryngitis/tracheitis (ALT), acute obstructive laryngitis/epiglottitis (AOLE), allergic rhinitis (AR), chronic rhinitis/nasopharyngitis (CRN), acute bronchitis (AB), chronic sinusitis (CS), asthma, other chronic obstructive pulmonary disease (COPD), and other diseases of hard tissues of teeth (DHTT).

This consistent approach to variable coding ensured uniformity and facilitated a thorough analysis of its relationship with AS.

### 2.3. Statistical Analysis

#### 2.3.1. Baseline Characteristics and Log-Rank Test

Baseline demographic and clinical characteristics were assessed using the initial recorded data of each participant in the dataset. Age was treated as a continuous variable and is presented as the mean with standard deviation. All other variables, being categorical, were summarized using frequency and percentage. The Log-Rank test, a non-parametric method suitable for comparing observed and expected events across groups over time, was employed to evaluate whether survival differences were statistically significant.

The test statistic was calculated using the following formula:

(1) $$X^{2}=\;\sum \frac{{\left(O_{i}-\;E_{i}\right)}^{2}}{E_{i}}$$

where *O_i_* represents the observed number of events in group *i*, and *E_i_* denotes the expected number of events assuming equal survival times across groups. Significant results suggest differences in survival curves between groups [22,23].

#### 2.3.2. Cox Proportional Hazards Model

Cox proportional hazards regression analyses were conducted to examine the association between the aforementioned respiratory conditions, related comorbidities, and the time to AS diagnosis. The Cox model was selected because it allows for the adjustment of multiple covariates without requiring specific assumptions about the survival distribution. This method produced outputs such as observed and expected events, hazard ratios (HRs), and corresponding *p*-values, offering insights into the independent impact of each variable on the risk of developing AS in the model.

The hazard function in the Cox model is given by:

(2) $$h\left(t\right)=\;h_{0}\left(t\right)\;exp(\beta_{1}X_{1}+\beta_{2}X_{2}+\cdots +\;\beta_{p}X_{p})$$

where *h*(*t*) represents the hazard at time *t*, *h*_0_(*t*) is the baseline hazard, and $\beta_{1},\;\beta_{2},\;\ldots ,\;\beta_{p}$ are the regression coefficients corresponding to covariates *X*_1_, *X*_2_, *…*, *X_p_*.

All predefined respiratory and comorbid covariates were retained in the multivariable Cox model, regardless of their prevalence, to preserve analytical scope and model stability.

We controlled for baseline covariates, including age and gender, to ensure that the HRs accurately reflected the effects of the examined predictors, independent of these demographic variables. The results of the Cox regression are reported as HRs with 95% confidence intervals (CIs). A hazard ratio above 1 indicates an increased risk, while a value below 1 suggests a protective effect [24,25].

#### 2.3.3. Model Validation and Assumption Testing

The Cox model’s reliability and strength were confirmed through multiple validation procedures. Initially, the proportional hazards assumption, crucial for the Cox model, was assessed using the Schoenfeld residual test. This test determines if the HRs for covariates remain stable over time; any inconsistencies might suggest a model misfit [26]. Additionally, linear predictions and predicted probabilities were computed to grasp the understanding of individual risk over time and to add clinical significance. The Schoenfeld residuals test verified that the HRs were stable over time, confirming the model’s suitability. Further robustness checks, such as subgroup analyses, were performed to ensure the findings’ stability and reliability.

#### 2.3.4. Model Performance Evaluation

To assess model discrimination, we performed a receiver operating characteristic (ROC) analysis and calculated the area under the curve (AUC) as a summary metric. ROC analysis evaluates the model’s sensitivity (true positive rate) and specificity (true negative rate) across various probability thresholds, offering critical insights into its predictive accuracy. Moreover, we computed Harrell’s C-index, which measures the concordance between predicted and observed events for all possible pairs in the dataset [27,28].

All statistical analyses and graphical outputs were executed using Stata software (version 18.0), with a significance threshold set at *p* < 0.05. This included the computation of survival and hazard estimates, as well as assessments of model fit and graphical diagnostics for comparison purposes [29].

## 3. Results

### 3.1. Study Population

At baseline, as further delineated in Table 1, the study comprised 4893 individuals, with a mean age of 44.52 years ± 15.50, ranging from 0 to 89 years. The sample was composed of 54.52% females and 45.48% males with low baseline prevalence of some factors.

In the analysis, 99.02% of participants did not get ANP [common cold], while 0.98% were affected. AP was present in 3.39% of the participants, with 96.61% not affected. AT was identified in 1.27% of the group, whereas 98.73% did not have this condition. ALT was observed in 1.12% of individuals, with 98.88% undocumented. AOLE was identified in 0.06% of the patients, while 99.94% were unremarkable. AR was manifested in 5.23% and lacking in 94.77%. CRN was underlined in 0.69% of the patients, with 99.31% not having the disease. AB was diagnosed in 3.88% of participants, with 96.12% remaining unaffected. CS was present in 0.61% of the sample, whereas 99.39% were not implicated. Asthma was detected in 2.49% of the patients, with 97.51% not presenting the condition.

Regarding comorbidities, 98.24% of participants did not have COPD, while 1.76% did. Additionally, other DHTT were exhibited in 6.64% and atypical in 93.36% of patients.

Out of 4893 patients, sex information was available for 4890 individuals. Three patients with missing sex data were excluded to ensure data integrity in the gender-stratified analysis. Apart from this, data for all respiratory and related comorbid conditions were available for the study population (4893 participants).

### 3.2. Factors Influencing Acute Sinusitis

Table 2 presents the results of the Log-Rank test, which assesses the equality of baseline survival functions by examining the impact of underlying parameters on survival duration.

The univariate survival analysis revealed that female sex and examined conditions, including ANP [common cold], AP, AT, ALT, AOLE, AR, CRN, AB, CS, asthma, other COPD, and other DHTT, were all significantly associated with shorter time to AS onset.

The Cox proportional hazards regression model was employed to assess the association between respiratory conditions, comorbidities, and the risk of AS. The findings are thoroughly presented in Table 3. This multivariate Cox model revealed that individuals with ANP [common cold] exhibited a 2.3-fold increased risk of developing AS compared to those without the condition (HR = 2.27, 95% CI [1.51–3.40], *p* < 0.001). Patients with AP demonstrated a 1.73-fold higher risk of AS (HR = 1.73, 95% CI [1.28–2.33], *p* < 0.001). Notably, AR was associated with the highest hazard, indicating a 4-fold increased risk (HR = 4.04, 95% CI [3.18–5.13], *p* < 0.001). Furthermore, episodes of AB elevated the risk of AS by 2.49 times (HR = 2.49, 95% CI [1.90–3.26], *p* < 0.001). Patients with CS experienced a 3.1-fold increase in AS risk (HR = 3.10, 95% CI [2.13–4.51], *p* < 0.001), whereas CRN did not exhibit a significant association with AS (HR = 1.41, 95% CI [0.92–2.18], *p* = 0.118). Although asthma was significantly associated with AS in the Log-Rank test (*p* < 0.001), it did not achieve statistical significance in the Cox regression model (HR = 1.04, 95% CI [0.71–1.54], *p* = 0.832). Importantly, comorbidities also significantly influence AS risk. Patients with COPD had a 1.6-fold increased risk of AS (HR of 1.62 [1.04–2.52], *p* = 0.031). Dental pathologies of hard tissues of teeth were linked to a 31% reduced risk of AS (HR of 0.69 [0.48–0.97], *p* = 0.034).

HR estimates for conditions with very small subgroups showed relatively wide CIs and warrant cautious interpretation.

### 3.3. Predictive Performance

**Figure 1:**
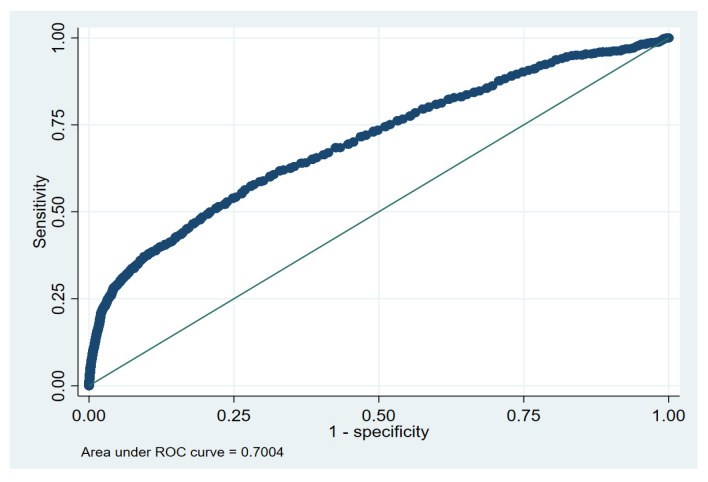
The Receiver Operating Characteristic (ROC) curve analysis. Note: ROC curve illustrated the trade-off between sensitivity and specificity for the Cox proportional hazards regression model.

The discriminative capability of the Cox regression model was assessed using Harrell’s C-index, which was 0.69, indicating moderate predictive accuracy. Model calibration and discrimination are further demonstrated in Figure 1.

## 4. Discussion

The primary objective of this study was to investigate a comprehensive array of respiratory and other comorbid conditions that may be linked to the risk of AS in clinical settings. By analyzing real-world data, this research provides evidence that explicit respiratory disorders and their related comorbidities clinically significantly increase the risk of AS.

The findings indicate that AR is a profound risk factor for AS. Both ANP [common cold] and CS were found to have a strong association with AS risk. Besides, it is noteworthy that AB, AP, along with COPD demonstrated high predictive values for the onset of AS diagnosis, whereas conditions such as CRN and various forms of acute laryngopharyngeal infections did not exhibit a significant association.

To the best of the authors’ knowledge, this is the first study to examine the predictive roles of both upper and lower respiratory factors combined with related comorbidities in the risk of developing AS by considering a wide range of conditions, accounting for patient demographic characteristics, and utilizing a large, well-documented study cohort. These findings are particularly relevant in the European context, where respiratory diseases, allergic conditions, and air pollution are among the major public health challenges [15,16].

One of the main findings of the study was the strong correlation between AR and AS diagnosis, emerging as a prominent predictor of AS, with a 4-fold increased risk observed in affected individuals. This association supports existing evidence that allergic inflammation contributes to sinonasal dysfunction, increasing susceptibility to infection [30,31]. The European Position Paper on Rhinosinusitis and Nasal Polyps highlights that allergic inflammation leads to nasal mucosal congestion, obstruction of sinus drainage pathways, and sinonasal microbiota dysbiosis, which in turn makes individuals more susceptible to bacterial colonization and sinus infections [16]. Research has demonstrated that AR can alter the sinonasal microbiota composition, fostering the proliferation of pathogenic bacteria while reducing the presence of beneficial commensal species. This issue is particularly pertinent in Hungary, where AR is widespread, affecting approximately 20–30% of the population. According to national statistics, its incidence is growing in urban areas due to factors such as air pollution and climate change [32]. Additionally, Hungary experiences elevated pollen levels, notably from ragweed, a common allergen that triggers seasonal exacerbations of AR [12]. In light of this association, improving the management of AR through the use of antihistamines, intranasal corticosteroids, allergen immunotherapy, or preventive nasal irrigations can help reduce the incidence and severity of AS. This supports the notion that untreated or inadequately managed AR may lead to recurrent sinus infections, underlining the necessity for focused monitoring and effective control of allergic diseases as part of AS prevention and management plans.

Another significant finding from our study was the 2.3-fold increased risk of AS in patients diagnosed with ANP, commonly referred to as the common cold. This observation corroborate the widely accepted principle that acute viral upper respiratory tract infections (URTIs) are a leading precursor to bacterial sinusitis [6]. The development of AS following a viral infection is well-documented, with virus-induced mucosal inflammation, increased mucus production, and impaired clearance mechanisms leading to secondary bacterial invasion [33]. The clinical practice guideline on sinusitis has shown that certain viral URTIs can progress to bacterial sinusitis, with common pathogens including *Streptococcus pneumoniae*, *Haemophilus influenzae*, and *Moraxella catarrhalis* [5]. Our findings highlight the position of early intervention in patients with ANP to prevent the onset of AS and assess its potential progression. In Hungary, seasonal viral infections, such as those caused by rhinovirus and influenza, are well-known triggers for URTIs, particularly during winter when temperature fluctuations and indoor crowding enhance viral transmission. The role of viral infections in predisposing individuals to AS is consistent with findings across Europe, where 0.5–2.0% of viral URTIs has the potential to develop to bacterial sinusitis [34]. This reflects the need for effective public health measures, including improved influenza vaccination coverage, equitable healthcare access, and appropriate availability of antiviral medications, to mitigate the risk of subsequent bacterial complications.

CS, in particular, was a significant factor in the risk of AS, with patients experiencing a 3.1-fold increase in risk, aligning with previous research that identifies chronic sinonasal inflammation as a key factor in recurrent bacterial infections [35]. CS is frequently associated with biofilm formation, which facilitates bacterial persistence and impedes the body’s immune defense mechanisms [16]. Structural abnormalities, such as nasal polyps and mucosal thickening, can also precipitate recurrent episodes of acute inflammation. In Hungary, chronic rhinosinusitis is particularly prevalent among individuals exposed to environmental pollutants, tobacco smoke, and occupational irritants [32]. According to data from the World Health Organization, Hungary exhibits one of the highest smoking rates in Europe, potentially exacerbating the prevalence of CS and the associated risk of AS [36]. Henceforth, comprehensive management strategies, including smoking cessation programs, education on nasal hygiene, and access to endoscopic sinus surgery for challenging cases are crucial to limit its virulence [37].

The findings also indicated a significant association between AB and AS events, with a 2.49-fold increased risk, supporting the unified airway concept, wherein inflammation in the respiratory tract can influence adjacent areas due to shared immune and anatomical connections [38]. The interaction between bronchial inflammation and sinonasal infection may arise from systemic inflammatory responses, impaired mucociliary clearance, and increased susceptibility to viral and bacterial infections in both the upper and lower airways [39]. Stevens WW et al. has further demonstrated that inflammatory mediators, such as interleukin-5 and eosinophils, impact both acute and chronic airway diseases, thereby reinforcing the connection between lower respiratory tract infections and sinus infections [40]. This highlights the importance of a holistic approach to managing respiratory diseases to prevent complications in both the upper and lower airways. In Hungary, respiratory infections, such as bronchitis, are prevalent during winter, particularly among children and the elderly [34]. The association between AB and AS may be attributed to systemic inflammatory responses, impaired mucociliary clearance, and increased susceptibility to secondary bacterial infections in the sinuses [41]. Moreover, the extensive use of antibiotics for respiratory infections has led to an intensified demand for antimicrobial stewardship programs to mitigate overuse and misuse, thereby addressing the chief issue of antibiotic resistance, an escalating public health concern in that nation [42].

Although asthma demonstrated a significant association with AS risk in univariate analysis, it lost significance in the multivariate Cox regression model. This suggests that while asthma may contribute to sinonasal inflammation, its standalone impact on AS risk could be influenced by other coexisting conditions, such as allergic rhinitis. Laidlaw TM et al. (2021) reported a strong connection between asthma and chronic rhinosinusitis, with both conditions sharing type 2 inflammatory pathways involving eosinophil activation and increased cytokine production [43]. However, the absence of a direct link with AS in our multivariate analysis suggests that asthma alone may not be a primary cause of acute bacterial sinus infections. In Hungary, the incidence of asthma is rising, with a major concern in urban regions where air pollution exacerbates airway inflammation [12]. The administration of inhaled corticosteroids in asthma management may alleviate the risk of AS by tackling underlying airway inflammation. Subsequently, further research is imperative to clarify the most plausible and precise mechanisms linking asthma and AS, especially in relation to various asthma phenotypes.

COPD was a considerable predictor of AS, consistent with studies indicating that COPD patients are more susceptible to upper respiratory tract infections due to chronic airway irritations and impaired mucociliary functions [44]. The accumulation of bacteria and systemic inflammation associated with COPD likely contribute to this heightened risk [45]. In agreement with Hungary’s high smoking rates and tangible burden of COPD, these findings emphasize the paramount importance of optimal respiratory care programs and ongoing health promotion initiatives.

Conversely, DHTT, conditions affecting the hard tissues of the teeth, appeared to exert a protective effect, which contrasts with studies linking odontogenic infections to maxillary sinusitis [46]. A possible explanation for this observation is that individuals who frequently receive dental care for hard tissue issues may maintain better oral hygiene, thereby reducing the risk of odontogenic sinus infections. Given the close anatomical and pathological links between the teeth and the paranasal sinuses, these findings underscore the potential impact of dental conditions on sinus disease and call the need for further focused research in this area.

Our findings indicated that age (HR = 0.98; 95% CI [0.98–0.98]; *p* < 0.001) was inversely related to the hazard of AS, suggesting that the risk of developing AS decreases with advancing age. Although the effect size was small, the narrow confidence interval and highly significant *p*-value suggest a consistent association. This observation aligns with a previous paper that identified a higher occurrence of AS in younger individuals, particularly children and young adults, compared to older adults [5]. The reduction in AS onset with advancing age may be attributed to various factors, such as changes in immune response, decreased exposure to upper respiratory infections, and anatomical or physiological changes in the nasal and sinus mucosa over time [6]. Young individuals, particularly children and adolescents, frequently encounter viral upper respiratory tract infections, which often precede bacterial AS [33]. Interestingly, older adults are less exposed to viral infections due to reduced interaction with large groups, such as schools and daycare centers, which diminishes the overall risk of secondary bacterial sinusitis. Additionally, recurrent infections in early life may lead to immune system adaptations that enhance mucosal defenses in adulthood. The reduced risk of AS in older adults may be related to differences in their healthcare-seeking behavior. On one hand, younger individuals, particularly those with children, are more likely to seek medical attention for respiratory symptoms, resulting in a higher reported incidence of AS. On the other hand, older adults may experience milder forms of AS or may be less inclined to seek medical care, potentially leading to an underestimation of the true prevalence of AS in these age groups [43]. These implications are especially applicable to the Hungarian healthcare system, where age-related disparities in respiratory healthcare utilization have been pronounced [42]. Younger populations may exhibit higher rates of medical consultations for respiratory illnesses, resulting in more diagnosed cases of AS. Meanwhile, older adults, particularly those with comorbidities, may present with atypical symptoms or have AS obscured by other chronic conditions, possibly causing underdiagnosis in this demographic.

The study also indicates that females have a significantly higher risk of developing AS compared to males. This finding is coherent with previous epidemiological studies suggesting that AS and other upper respiratory conditions are more prevalent in women than in men [16,38]. Several biological and behavioral factors may account for this females’ increased susceptibility. Hormonal influences are prominent, as estrogen affects nasal mucosal function, immune responses, and inflammatory processes, markedly increasing the vulnerability to sinus infections in women [47]. Previous research suggested that changes in estrogen levels, particularly during menstruation, pregnancy, and menopause, can influence nasal obstruction and mucosal swelling, making females more prone to recurrent sinus infections [48]. Additionally, variations in immune function between genders may explain this elevated risk. Women generally exhibit stronger innate and adaptive immune responses than men, which, while beneficial in combating pathogens, can also lead to increased inflammatory responses and a higher likelihood of developing sinus-related issues [49]. This exaggerated response may contribute to prolonged or intensified inflammation in the nasal and sinus mucosa, increasing the likelihood of AS. Beyond biological factors, dissimilarities in healthcare services’ utilization between the sexes may also significantly contribute to the manifested higher likelihood of AS. This trend is particularly common among women, who tend to seek medical care more frequently than men for disease symptoms, leading to a higher reported incidence of AS in females [50,51]. This is parallel to conclusions from Hungarian healthcare studies, where women have been shown to have higher consultation rates and prescriptions for respiratory diseases than men [42]. The implications of this gender disparity are central for public health planning and clinical implementation. With respect to the increased risk of AS in females, healthcare professionals should pay utmost vigilance to the diagnosis and management of AS in women, especially those with concurrent risk factors such as AR, COPD, or other hormonal fluctuations. Targeted awareness campaigns and preventive measures, including at-risk female populations, are likely to mitigate the burden of AS and reduce healthcare costs associated with recurrent sinonasal infections.

Our research partially coincides with the COVID-19 period (March 2020 to December 2022). Similar to other countries, this crisis overwhelmed Hungary and caused significant disruptions in the respiratory disease landscape. The study encountered challenges in accurately assessing the impact of COVID-19 infections due to incomplete data or limited information on confirmed cases in our clinical dataset, especially during the early stages of the pandemic. However, as reported by the National Center for Public Health in Hungary [52], there was a partial decline in non-COVID respiratory diagnoses during this period. This decline was attributed to the success of mandatory syndromic surveillance through lockdowns, phased restrictions, the vaccine-certificate system, and social distancing measures. Additionally, the low rate of active infections closely reflected the level of previous exposure to severe acute respiratory syndrome coronavirus 2, indicating that the containment measures during the pandemic might have effectively limited transmission, as presented in a nationwide population-based study in Hungary [53]. Besides, future investigations, applying segmented analytical or time-series designs, could more accurately delineate how the COVID-19 pandemic and related public health interventions modified the association between respiratory comorbidities and the risk of AS.

This study has several notable strengths. It drew on a large real-world clinical dataset, allowing for detailed statistical analyses and subgroup comparisons. Diagnoses were based on physician-assigned ICD-10 codes, enhancing diagnostic accuracy and clinical relevance. The well-documented 15-year follow-up period further provided meaningful insights into the long-term risk patterns and progression of AS over time.

Despite its strengths, this research encountered certain limitations. Firstly, reliance on electronic health records might have introduced some biases due to diagnostic coding errors or incomplete data. Secondly, the study population was derived from a single clinical center, possibly limiting the generalizability of the findings. Thirdly, the study did not account for some potential confounders, such as environmental exposures (e.g., aeroallergen hazards) or other related factors (e.g., smoking as a lifestyle indicator), which could influence AS risk development. Smoking, a significant confounding factor, was not included in the analysis due to the absence of smoking habits or status in the clinical database. Nonetheless, we believe this limitation was addressed as the effect of COPD was indirectly considered in our model. Additionally, anatomical variations affecting sinonasal ventilation and drainage, such as nasal septal deviations, along with records of facial trauma, sinonasal surgery, or interventions disrupting sinus anatomy, were not systematically captured in health records. Our analysis did not model seasonality or stratify by season; therefore, preventing assessment of temporal trends. The study’s partial overlap with the COVID-19 era likely altered healthcare-seeking behavior and diagnostic coding practices for respiratory diseases. These disruptions may have impacted observed patterns of AS and comorbidities, which could not be fully disentangled in this analysis. Finally, the retrospective design may preclude the establishment of causal links between exposure and outcome.

Future research should verify these findings in diverse populations and ultimately discover the potential role of different biomarkers, genetic predispositions, or precision medicine in predicting AS risk. Notably, further analyses are needed to address the limitations of this study by incorporating multicenter cohorts and other detailed covariates. In particular, prospective longitudinal studies are demanded to elucidate a nuanced understanding of temporal relationships beyond estimated associations and their impact on broader populations.

## 5. Conclusions

Patients with AR, CS, AB, ANP [common cold], or COPD are at markedly increased risk of developing AS. Routine clinical assessment should include screening for these underlying conditions in individuals presenting with recurrent or severe respiratory symptoms. Proactive management of allergic and chronic airway diseases, along with optimized control of smoking-related lung disorders, may substantially reduce the frequency and severity of AS episodes. Integrating allergy care, smoking cessation, and rational antibiotic use into standard respiratory care pathways represents a practical and effective approach to lowering sinusitis overall burdens, minimizing its complications, and improving patient outcomes.

## Figures and Tables

**Table 1:** Baseline characteristics of the study population at the initiation of the follow-up period.

Variables	Categories	*n* (%)	Total *n* *
Age	Mean (SD) Min–Max	44.52 (15.50) 0–89	4893
Gender	Male	2224 (45.48%)	4890
	Female	2666 (54.52%)	
Acute nasopharyngitis [common cold]	No	4845 (99.02%)	4893
	Yes	48 (0.98%)	
Acute pharyngitis	No	4727 (96.61%)	4893
	Yes	166 (3.39%)	
Acute tonsillitis	No	4831 (98.73%)	4893
	Yes	62 (1.27%)	
Acute laryngitis/tracheitis	No	4838 (98.88%)	4893
	Yes	55 (1.12%)	
Acute obstructive laryngitis/epiglottitis	No	4890 (99.94%)	4893
	Yes	3 (0.06%)	
Allergic rhinitis	No	4637 (94.77%)	4893
	Yes	256 (5.23%)	
Chronic rhinitis/nasopharyngitis	No	4859 (99.31%)	4893
	Yes	34 (0.69%)	
Acute bronchitis	No	4703 (96.12%)	4893
	Yes	190 (3.88%)	
Chronic sinusitis	No	4863 (99.39%)	4893
	Yes	30 (0.61%)	
Asthma	No	4771 (97.51%)	4893
	Yes	122 (2.49%)	
Other chronic obstructive pulmonary diseases	No	4807 (98.24%)	4893
	Yes	86 (1.76%)	
Other diseases of hard tissues of teeth	No	4568 (93.36%)	4893
	Yes	325 (6.64%)	

* *n* represents the study population. Unless otherwise specified, all values are presented as number (percentage) [*n* (%)].

**Table 2:** Outcomes of the Log-Rank test conducted to compare survival distributions according to baseline parameters.

Variables	Categories	Observed Events	*p*-Value *
Gender	Male	288	**0.01**
	Female	524	
Acute nasopharyngitis [common cold]	No	781	**<0.001**
	Yes	31	
Acute pharyngitis	No	743	**<0.001**
	Yes	69	
Acute tonsillitis	No	791	**<0.001**
	Yes	21	
Acute laryngitis/tracheitis	No	793	**<0.001**
	Yes	19	
Acute obstructive laryngitis/epiglottitis	No	810	**<0.001**
	Yes	2	
Allergic rhinitis	No	691	**<0.001**
	Yes	121	
Chronic rhinitis/nasopharyngitis	No	787	**<0.001**
	Yes	25	
Acute bronchitis	No	739	**<0.001**
	Yes	73	
Chronic sinusitis	No	778	**<0.001**
	Yes	34	
Asthma	No	774	**<0.001**
	Yes	38	
Other chronic obstructive pulmonary diseases	No	785	**<0.001**
	Yes	27	
Other diseases of hard tissues of teeth	No	779	**0.004**
	Yes	33	

* Values in bold indicate statistical significance (*p* < 0.05) as determined by the Log-Rank test.

**Table 3:** Results from the Cox proportional hazards regression analysis.

Variables (ICD-10 Codes)	Categories (Reference)	HR [95% CI]	*p*-Value *
Age	Continuous	**0.98 [0.98–0.98]**	**<0.001**
Gender	Female (ref: Male)	**1.18 [1.02–1.36]**	**0.027**
Acute nasopharyngitis [common cold] (J00)	Yes (ref: No)	**2.27 [1.51–3.40]**	**<0.001**
Acute pharyngitis (J02)	Yes (ref: No)	**1.73 [1.28–2.33]**	**<0.001**
Acute tonsillitis (J03)	Yes (ref: No)	0.90 [0.55–1.46]	0.666
Acute laryngitis/tracheitis (J04)	Yes (ref: No)	0.97 [0.59–1.59]	0.901
Acute obstructive laryngitis/epiglottitis (J05)	Yes (ref: No)	1.49 [0.33–6.60]	0.603
Allergic rhinitis (J30)	Yes (ref: No)	**4.04 [3.18–5.13]**	**<0.001**
Chronic rhinitis/nasopharyngitis (J31)	Yes (ref: No)	1.41 [0.92–2.18]	0.118
Acute bronchitis (J20)	Yes (ref: No)	**2.49 [1.90–3.26]**	**<0.001**
Chronic sinusitis (J32)	Yes (ref: No)	**3.10 [2.13–4.51]**	**<0.001**
Asthma (J45)	Yes (ref: No)	1.04 [0.71–1.54]	0.832
Other chronic obstructive pulmonary diseases (J44)	Yes (ref: No)	**1.62 [1.04–2.52]**	**0.031**
Other diseases of hard tissues of teeth (K03)	Yes (ref: No)	**0.69 [0.48–0.97]**	**0.034**

* Values in bold denote statistically significant associations (*p* < 0.05). Abbreviations: HR, Hazard Ratio; CI, Confidence Interval. The reported hazard ratios were adjusted for other variables included in the model. The area under the curve (AUC) was 0.71 (95% CI [0.68–0.72]).

## Data Availability

The dataset underpinning this research can be accessed by directly contacting the corresponding author of this study. Owing to institutional policies, the data cannot be made publicly available.

## Abbreviations

The following abbreviations are used in this manuscript:

AB	Acute Bronchitis
ALT	Acute Laryngitis/Tracheitis
ANP	Acute Nasopharyngitis [Common Cold]
AOLE	Acute Obstructive Laryngitis/Epiglottitis
AP	Acute Pharyngitis
AR	Allergic Rhinitis
AS	Acute Sinusitis
AT	Acute Tonsillitis
COPD	Chronic Obstructive Pulmonary Disease
CRN	Chronic Rhinitis/Nasopharyngitis
CS	Chronic Sinusitis
DHTT	Diseases of Hard Tissues of Teeth
